# Multimodal Temperature Readout Boosts the Performance of CuInS_2_/ZnS Quantum Dot Nanothermometers

**DOI:** 10.1021/acsami.4c14541

**Published:** 2024-10-22

**Authors:** Magdalena Duda, Pushkar Joshi, Anna Borodziuk, Kamil Sobczak, Bozena Sikora-Dobrowolska, Sebastian Maćkowski, Allison M. Dennis, Łukasz Kłopotowski

**Affiliations:** † Institute of Physics, Polish Academy of Sciences, 02-668 Warsaw, Poland; ‡ University of Warsaw Biological and Chemical Research Centre, 02-089 Warsaw, Poland; § Institute of Physics, Faculty of Physics, Nicolaus Copernicus University, Astronomy and Informatics, 87-100 Toruń, Poland; ∥ Department of Chemical Engineering, Northeastern University, Boston, Massachusetts 02115, United States

**Keywords:** nanothermometers, photoluminescence, quantum dots, multiple linear regression, CuInS_2_, optical sensors, luminescent nanomaterials

## Abstract

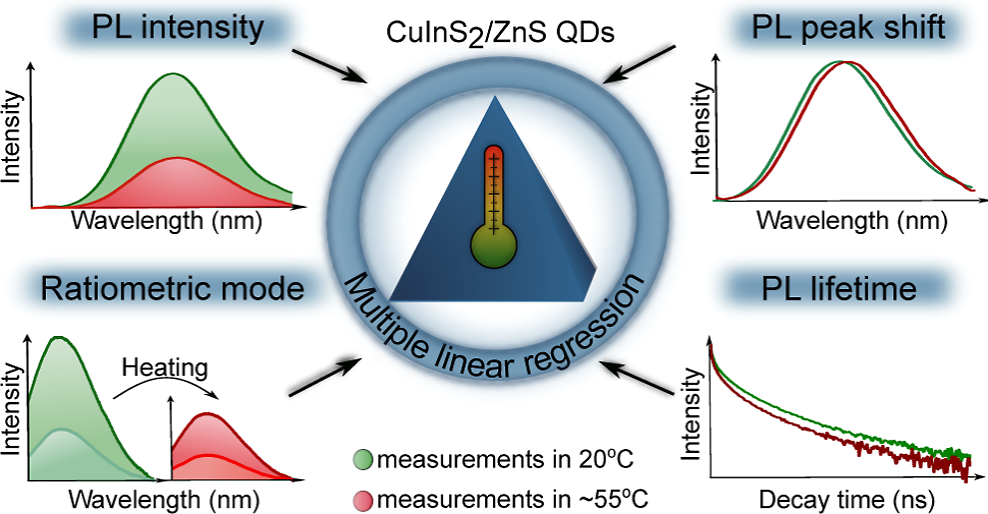

Fluorescent nanothermometers are positioned to revolutionize research into cell functions and provide strategies for early diagnostics. Fluorescent nanostructures hold particular promise to fulfill this potential if nontoxic, stable varieties allowing for precise temperature measurement with high thermal sensitivities can be fabricated. In this work, we investigate the performance of micelle-encapsulated CuInS_2_/ZnS core/shell colloidal quantum dots (QDs) as fluorescent nanothermometers. We demonstrate four temperature readout modes, which are based on variations in the photoluminescence intensity, energy, and lifetime and on a specific ratio of excitation efficiencies. We further leverage this multimodal readout to construct a fifth, multiparametric thermometer calibration based on the multiple linear regression (MLR) model. We show that the MLR approach boosts the thermometer sensitivity by up to 7-fold while reducing the readout error by about a factor of 3. As a result, our QDs offer the highest sensitivities among semiconducting QDs emitting in the first biological window. The obtained results indicate that CuInS_2_/ZnS QDs are excellent candidates for intracellular in vivo thermometry and provide guidelines for further optimization of their performance.

## Introduction

Fluorescent nanothermometers are nanostructures exhibiting temperature-dependent fluorescence properties that can be deployed to monitor the temperature of their environment.^1^ The development of fluorescent nanothermometers has enabled hitherto impossible studies of cell functions, revealing effects calling for a critical reexamination of the role of mitochondria in thermogenesis^2^ and identifying areas for future research of metabolic diseases.^3^ Monitoring the cell temperature in vivo enables research demonstrations of early tumor detection,^4^ diagnosis of early stages of ischemia,^5^ visualization of brain activity,^6^ and the mapping of heterogeneous heat production,^7^ to cite a few milestones. Intracellular temperature sensors are also crucial for monitoring the heat distribution upon application of hyperthermia therapies in order to avoid damage to healthy tissues surrounding the malignant ones.^8^ Notably, fluorescent nanothermometers are also employed for monitoring temperature in microelectronic devices,^9^ microfluidic devices,^10^ and catalysis.^11^

The above achievements notwithstanding, currently available nanothermometers all suffer from disadvantages that limit their functionality. Organic nanothermometers, i.e., fluorescent polymers,^12^ fluorescent proteins,^13^ or fluorescent dyes,^14^ exhibit excellent biocompatibility but suffer from strong photobleaching; their stability varies depending on the structure, and, in general, they exhibit relatively low thermal sensitivities.^15^ Lanthanide-doped upconverting nanoparticles (UCNPs) are optically and structurally stable but require high excitation densities to achieve intense upconverted photoluminescence (PL), contain heavy and rare elements, and exhibit intrinsically low brightness.^16^ Semiconductor quantum dots (QDs) are robust against photobleaching and offer size- and temperature-dependent optical properties. However, the sensitivities of QD nanothermometers remain smaller than for the competing materials,^17^ and the most widely studied compounds are cadmium and lead chalcogenides. The application of these compounds in any biological thermal imaging is hindered by the inherent toxicity of Pb^2+^ and Cd^2+^ ions, which are released to the environment upon QD degradation.^18^ As a consequence, a lot of research effort is devoted to finding alternative, Cd- and Pb-free QD materials.^19^

One of such materials is carbon dots. These nanostructures can be easily synthesized by using a variety of low-cost precursors. Carbon dots exhibit excellent biocompatibility, easy surface biofunctionalization, tunable optical properties, and high thermal sensitivities.^20^ However, the PL spectra are usually strongly dependent on the excitation conditions, complicating applications and comparison of results, and the photostability of carbon dots is questionable and strongly sample-dependent.^21^ Moreover, the structure–property relationship as well as the PL mechanism in carbon dots is a matter of an ongoing debate^22^ hindering further development. Another alternative to Cd and Pb chalcogenides is Ag_2_S QDs, which offer broadband absorption and stable PL in the near-infrared range (second biological window).^23^ However, Ag_2_S QDs still suffer from low brightness,^24^ and understanding of photothermal effects is incomplete.^25^

In this work, we focus on another material. CuInS_2_/ZnS (CIS/ZnS) core/shell QDs are alternatives that have been proven photostable and significantly less toxic than Cd- or Pb-containing QDs.^26−28^ Absorption cross sections of CIS/ZnS QDs are similar in magnitude to CdSe QDs,^29^ and core/shell architectures enable PL quantum yields (QYs) approaching unity.^30,31^ The high brightness of CIS/ZnS QDs and PL lifetimes longer than 200–300 ns^29,32,33^ make them exciting emitters for bioimaging.^34^ Initial studies on CIS/ZnS QDs as fluorescent nanothermometers have already appeared.^35,36^ Marin and co-workers developed a temperature readout based on deconvolution of the PL peak into three temperature-dependent components. In another study, Zhang and co-workers demonstrated a temperature readout based on the changes in the total PL intensity. The reported sensitivities were in the range of 1–2% °C^–1^, on par with other QD systems but significantly lower than, e.g., codoped dielectric phosphors.^37,38^ Moreover, both of the reported temperature readout modes are contingent on PL intensity variations and therefore prone to substantial errors resulting from the inhomogeneous distribution of QDs inside the studied medium. It is clear that employing CIS/ZnS QDs in intracellular nanothermometry requires the development and optimization of new readout modes, providing higher readout sensitivities.

In this work, we study temperature-dependent PL properties of aqueous suspensions of micelle-encapsulated CIS/ZnS QDs. We demonstrate temperature readout based on (i) PL intensity, (ii) PL lifetime, and (iii) PL peak position and a readout performed in a (iv) ratiometric mode, in which the ratio of PL intensities excited at two specific wavelengths encodes the temperature. We evaluate the thermometer sensitivities and find values comparable to those of other QD systems. Crucially, the presence of four independent readout modes enables combining them into a multiparametric readout based on multiple linear regression (MLR).^39,40^ The MLR-based readout provides sensitivities up to 7 times higher than those obtained using single parameters. As a result, CIS/ZnS-based nanothermometers reported here exhibit the highest sensitivities among all QD materials emitting in the first biological window. The MLR approach also decreases the readout error by about a factor of 3. Our results indicate that CIS/ZnS QDs are excellent markers for intracellular, in vivo temperature sensing.

## Results and Discussion

### Description of Samples

Experiments were performed on a series of CIS/ZnS core/shell QD samples, where the duration of the shelling reaction varied from 0 to 150 min. Hereafter, we label the samples with the shelling time, i.e., CIS/ZnS-0, CIS/ZnS-30, etc. The synthesis protocol was based on refs (29, 41, and 42), see the Materials and Methods section. QDs synthesized with this method have a pyramidal shape (see the Supporting Information, Figure S1) and chalcopyrite crystal structure as shown in refs (29, 33, and 41). To evaluate the mean size of the core-only CIS/ZnS-0 QDs, we determined the position of the first excitonic absorption transition from the analysis of the second derivative of the absorption spectrum (see the Supporting Information, Figure S2 for optical characterization of the studied samples). The mean size of 2.3 nm was evaluated using the sizing curve reported by Xia et al. in (43). Overgrowth of the CIS cores with ZnS resulted in a blue shift of the absorption edge and PL peak, both a consequence of the interplay between core etching, Zn alloying into the core, and heteroepitaxial formation of a ZnS shell.^44^ The process also resulted in an increased QY from 2% for CIS/ZnS-0 to 24% for CIS/ZnS-60 (see the Supporting Information, Figure S2E). Similar results can be found in the literature.^42,45^ As-obtained QDs were hydrophobic owing to the presence of dodecanethiol (DDT) ligands on the surface of CIS/ZnS-0 and DDT and oleic acid (OA) ligands on the shelled QDs. In order to study the temperature-dependent properties in an environment compatible with the target application of intracellular nanothermometers, transfer to an aqueous environment was necessary. To this end, we encapsulated the QDs in an amphiphilic polymer^46^ using the procedure of Zhang et al.^36^ Encapsulation and transfer to water resulted in a small red shift of the PL peak originating from the change of the electron–hole Coulomb interaction induced by the change of the solvent polarity (see the Supporting Information, Figure S3).^35,36^ Importantly, as we showed in previous work, the micellization decreased the PL QY only by a factor of about 2.^47^ The results of the stability study of the micelle-encapsulated QDs are shown in the Supporting Information, Figure S4. We found negligible loss of PL intensity during the first ∼20 days. After 56 days, the PL intensity decreased to 80% of the initial value. Over this time period, the change in absorbance was negligible, indicating insignificant structural degradation of the micelle-encapsulated CIS/ZnS QDs.

### Modes of Temperature Readout

**Figure 1:**
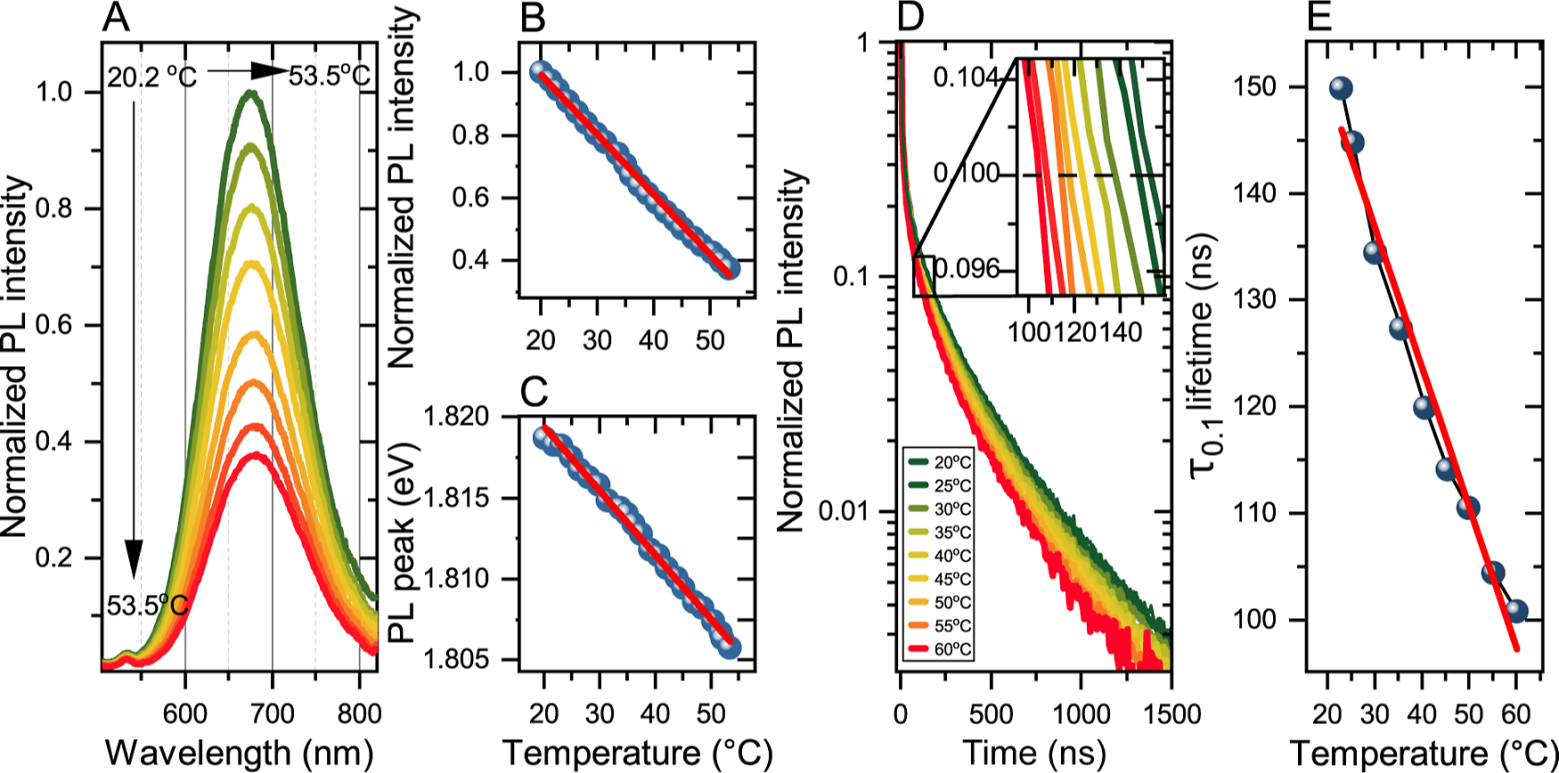
(A) PL spectra excited at 450 nm measured as a function of temperature for CIS/ZnS-30 QDs encapsulated in micelles. (B) Temperature dependence of the PL intensity normalized to the value at 20 °C (points) and fitted linear function is *Q*_I_(*T*) = −0.0191*T* + 1.37 (line). (C) PL peak position as a function of temperature (points) and fitted linear function *Q*_E_(*T*) = −0.00039*T* + 1.83 (line). (D) Temperature dependence of normalized PL decays excited at 400 nm. (E) Temperature dependence of PL lifetime (blue points). The lifetime is defined as the decay time at which the intensity drops by a factor of 10—see the inset in (D). The lifetimes are fitted with a linear function *Q*_τ_(*T*) = −1.31*T* + 176 (red line).

Operation of three modes of the temperature readout from CIS/ZnS QDs is presented in Figure 1. The results are shown for CIS/ZnS-30. The remaining samples exhibit qualitatively the same behavior (see Supporting Information, Figures S5–S7). Quantitative differences between the samples are discussed below. Temperature dependence of the PL spectra of sample CIS/ZnS-30 is presented in Figure 1A. The spectra are broad with full width at half-maximum (fwhm) line widths larger than 200 meV—a common feature of CIS-based QDs and a consequence of a strong electron–phonon coupling combined with size inhomogeneity.^29,33,35,36,41,45^ Clearly, as the temperature is increased, the PL intensity decreases. We attribute this effect to thermal activation of nonradiative decay channels via carrier trapping at surface states.^33,45,48^ The temperature-dependent integrated PL intensity *I*_PL_(*T*) normalized to the value at 20 °C is plotted in Figure 1B. As the temperature is increased from 20 to 54 °C, we observe that the PL intensity decreases by around 63%. The PL intensity quenching with temperature enables the temperature readout by evaluating the normalized intensity *Q*_I_(*T*) = *I*_PL_(*T*)/*I*_PL_(20 °C). Note that nanothermometer operation based on *Q*_I_ was demonstrated previously in ref (36), where a smaller quenching rate was reported (see the discussion below).

Analysis of the PL spectra in Figure 1A shows that an increase in temperature results also in a red shift of the PL peak (normalized PL spectra are presented in Figure S8 in the Supporting Information). We attribute the shift to closing of the semiconductor band gap with temperature. The PL peak position as a function of temperature, *E*_PL_(*T*), is plotted in Figure 1C. In the studied temperature range, the PL peak shifts by ∼13 meV, which corresponds to a rate d*E*_PL_/d*T* ≈ – 0.39 meV/°C. Thus, the second mode of temperature readout is based on the evaluation of *Q*_E_ = *E*_PL_(*T*). This mode is obviously insensitive to concentration variations and offers the possibility of evaluating the absolute temperature. We note, however, that compared to the PL fwhm line width, which for CIS/ZnS-30 is 350 meV, the temperature-induced shift is small, so this mode is expected to suffer from low precision unless very strong PL signals are measured.^49^

The third mode of temperature readout is based on the changes in PL lifetime. PL decays measured as a function of temperature for sample CIS/ZnS-30 are presented in Figure 1D. The decays are multiexponential with long components in the μs range as shown by many reports.^29,33,41,45^ As the temperature is increased from 23 to 60 °C, we observe an acceleration of the PL decay. This effect is another manifestation of thermal activation of nonradiative recombination. To avoid random errors related to fitting multiexponential decays with weak, long-lived components, we define lifetime τ_0.1_ as the time delay at which the intensity decreases 10-fold with respect to the initial value. A similar approach was employed in ref (32). The values of τ_0.1_ are plotted as a function of temperature in Figure 1E. We observe a clear shortening of τ_0.1_ from 150 ns at 23 °C to 101 ns at 60 °C. Therefore, in this mode, the temperature readout is established by evaluating *Q*_τ_(*T*) = τ_0.1_(*T*). In the Supporting Information, Figure S4C and D, we demonstrate the reversibility of the temperature-induced changes to PL intensity and peak position. Furthermore, the stability of the PL intensity and PL lifetime under continuous UV excitation is demonstrated in Figure S4E,F.

The three readout modes presented above are commonly reported for fluorescent nanothermometers based on semiconductor QDs.^50−53^ Another convenient mode providing concentration-independent results is a ratiometric mode, in which a PL intensity ratio of two peaks encodes the temperature.^15^ This mode is an asset of nanothermometers based on lanthanide-doped UCNPs, in which the occupation of specific emissive dopant states is governed by Boltzmann statistics.^54^ The availability of this mode resulted in a plethora of reports on applications of UCNPs in biomedicine and beyond.^1,55^ To implement a ratiometric temperature readout from a QD-based nanothermometer, a dual-emitting architecture can be employed by introducing emissive dopants,^56^ by overgrowing the QD core with an emissive shell,^50^ or by otherwise singling out a distinct emission band,^57,58^ thereby providing a second emission channel operating alongside the core PL.

In this work, we decided to pursue a different approach. Namely, we analyzed the temperature dependence of the PL intensity excited with two excitation wavelengths, thereby creating carriers significantly differing in excess energy with respect to the PL energy. We note that a similar strategy was successfully employed to achieve a ratiometric temperature readout from excitation intensity ratio in green fluorescent protein^59^ and Nd/Cr codoped phosphors.^60^

**Figure 2:**
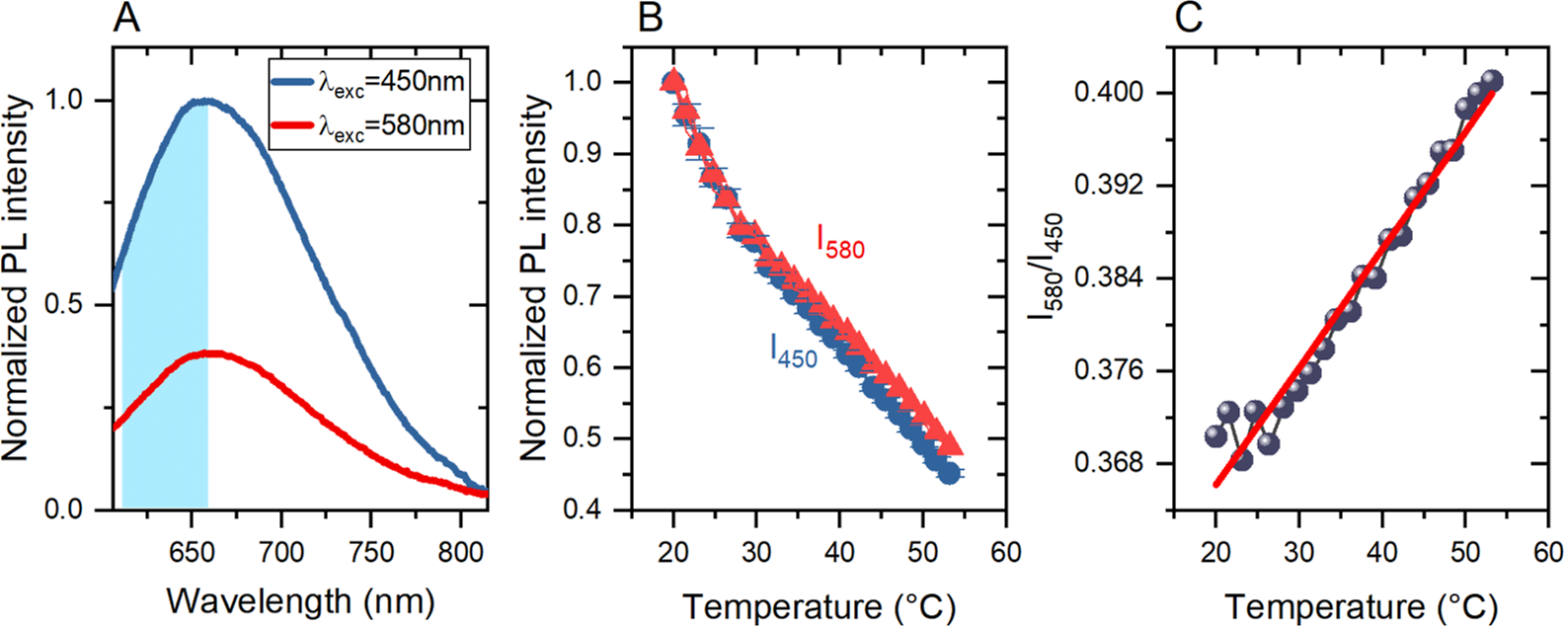
(A) PL spectra after 450 (blue line) and 580 nm (red line) excitation. The highlighted area denotes the spectrum integration range. (B) Normalized integrated PL intensities as a function of temperature, for excitation at 450 nm (*I*_450_, blue circles) and 580 nm (*I*_580_, red triangles). (C) Temperature dependence of *I*_450_/*I*_580_ intensity ratio (blue points) and fitted linear function is *Q*_R_(*T*) = 0.00102*T* + 0.35 (red line).

In Figure 2A, we plot the PL spectra of sample CIS/ZnS-30 recorded at 20 °C for two excitation wavelengths: 450 and 580 nm. For these two spectra, the intensity ratio *I*_580_/*I*_450_ is 0.38, while the ratio of the number of absorbed excitation photons is (1–10^–*A*(580)^)/(1–10^–*A*(450)^) = 0.25, where *A*(λ_ex_) is the absorbance at the excitation wavelength λ_ex_. Thus, *I*_580_ is larger than that expected from the wavelength dependence of absorption. The above discrepancy indicates that the PL QY depends on the excitation energy. Such dependence has been previously reported for CdSe QDs^61,62^ and, very recently, for CIS/ZnSSe core/shell QDs.^31^ In both cases, the PL QY decreased with an increase in the excitation energy (decreasing wavelength). In agreement with these reports, the result presented in Figure 2A indicates a lower PL QY upon higher excitation energy (shorter wavelength). Because of the excitation energy dependence of the PL QY, the *I*_580_/*I*_450_ ratio is determined not only by the absorption strength but also by different relaxation efficiencies from the excited to the luminescent state when exciting at the two wavelengths. Following the conclusions of ref (61), we attribute the stronger PL quenching for higher excitation energies to more efficient hot carrier trapping, competing with the relaxation toward the luminescent state. For further discussion of the physical mechanisms behind the temperature-dependent optical properties, see the Supporting Information, Section S5 and Figure S12.

The presence of two distinct relaxation channels provides an indication that a ratiometric temperature readout can be possible if the two relaxation efficiencies exhibit a different temperature dependence. In Figure 2B, we plot the temperature dependence of *I*_580_(*T*) and *I*_450_(*T*), normalized to their values at *T* = 20 °C. Clearly, the PL quenching process occurs at different rates for the two cases. In Figure 2C, we plot the intensity ratio *Q*_R_(*T*) = *I*_580_(*T*)/*I*_450_(*T*). A definite trend can be observed as *Q*_R_(*T*) increases with the temperature. This behavior allows us to determine a fourth mode of temperature readout based on *Q*_R_. The results for the other samples are similar and are presented in Supporting Information, Figures S9–S11. Since both the PL and absorbance spectra change with ZnS shelling time, we consistently choose the excitation wavelengths to enable comparison of thermometer sensitivities between the samples. Namely, for each sample, the short excitation wavelength was fixed at 450 nm, while the long excitation wavelength was chosen such that the ratio between the absorbance at 450 nm and the long wavelength was equal to 5.5. The chosen excitation wavelengths are given in the Supporting Information and Table S1.

### Comparison of Nanothermometers with Different Shelling Times

To compare the nanothermometer performance of QDs with different shelling times and compare the performance of the readout modes, for each sample and mode, we evaluate the relative sensitivity as^1,15^
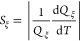
1where ξ is *I*, *E*, τ, or *R*.

**Figure 3:**
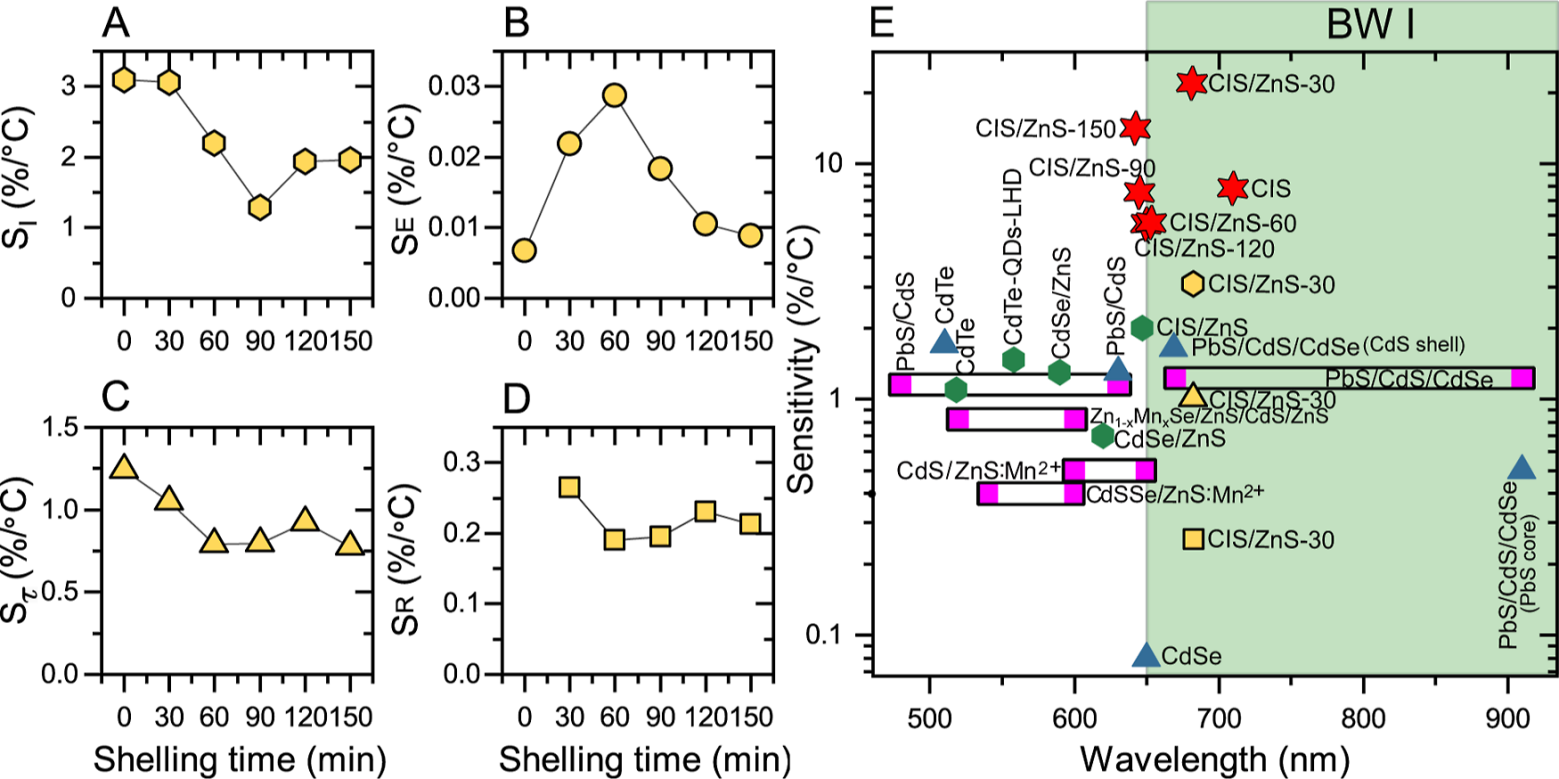
(A–D) Thermal sensitivities of the four single-parameter readout modes for samples with different ZnS shelling times. Readout is based on (A) PL intensity, (B) PL peak shift, (C) PL lifetime, and (D) PL intensity ratio after excitation at two wavelengths. (E) Comparison of sensitivities plotted as a function of emission wavelength for different QD systems. Red stars denote the sensitivity values from the multiparametric, MLR-based readout. The triangles, hexagons, and squares denote the sensitivity values for QD nanothermometers based on PL lifetime, PL intensity, and a ratiometric mode, respectively. Yellow symbols represent the highest sensitivity values obtained with the QDs studied in this work. Data presented for other QDs were extracted from refs (36, 50−52, and 63−68). The highlighted green area marks the first biological window (BW I).

In Figure 3A–D, we present the values of *S*_ξ_ averaged over the studied temperature range as a function of ZnS shelling times for the four temperature readout modes. We obtain the highest sensitivities for the readout mode based on the intensity quenching *Q*_I_. The highest values of *S*_I_, see Figure 3A, are equal to 3.1% °C and are obtained for the core-only CIS QDs and core/shell CIS/ZnS-30. This value is in fact larger than *S*_I_ for other QD-based nanothermometers, including CIS/ZnS,^35,36^ found in the literature (see the comparison presented in Figure 3E and Table S2 in the Supporting Information). As the shelling time increases, *S*_I_ decreases along with the increase of PL QY (compare Figures 3A and S2E). Therefore, we attribute the decrease of *S*_I_ to progressively stronger blocking of carrier trapping and a weaker activation of nonradiative recombination. Both of these effects are expected as a consequence of the growth of an increasingly thicker ZnS shell.

The sensitivity of the readout mode based on the energy position of the PL peak manifests a nonmonotonic dependence, see Figure 3B. For the core-only CIS/ZnS-0 sample, *S*_E_ is negligible. This is expected since the band gap of bulk CuInS_2_ exhibits a weak temperature dependence, a consequence of the fact that the valence-to-conduction band transition mainly couples states of the sulfur sublattice.^69^ The sensitivity is increased to 0.022% °C for CIS/ZnS-30 and 0.029% °C for CIS/ZnS-60 and decreases with further increasing the shelling time to about 0.01% °C. Although the highest evaluated sensitivities are significantly larger than for CdSe QDs (see the Supporting Information, Table S3), we expect that the precision of the readout with this mode will be low because the temperature-induced shift is small compared to the PL line width.^49^

The shelling time dependence of the nanothermometer sensitivity with the readout based on the PL lifetimes is shown in Figure 3C. For CIS/ZnS-0 and CIS/ZnS-30, we achieve sensitivities of about 1% °C. This value is similar to *S*_τ_ reported for PbS/CdS/CdSe QDs and, remarkably, is higher than other competing QD structures emitting in the first biological window (650–950 nm). Similar to *S*_I_, the values of *S*_τ_ roughly decrease with increasing shelling time. As discussed above, we attribute this effect to a weaker activation of nonradiative recombination due to the formation of a thicker ZnS shell. For a detailed comparison of the QD-based nanothermometer sensitivities operating by measurement of the PL lifetimes, see Supporting Information and Table S4.

The sensitivities of the CIS/ZnS QD nanothermometers based on the ratiometric readout mode are shown in Figure 3D. The obtained values lie between 0.19 and 0.26% °C which make them comparable to sensitivities of widely studied ratiometric nanothermometers based on Nd-, Tm-, Er-, and/or Yb-doped UCNPs emitting in the first biological window. We note, however, that the obtained values are slightly lower than for semiconducting QDs with a readout based on emission ratio (see Supporting Information and Table S5). Further optimization of the excitation wavelengths is necessary to determine the sensitivity limits of this readout mode.

In general, the comparison presented in Figure 3E allows us to conclude that CIS/ZnS QD nanothermometers operating via single-parameter readouts yield sensitivities comparable to those reported for other systems. In particular, the strong temperature dependence of the PL intensity and PL lifetime makes the modes based on these parameters promising for intracellular sensing.

Relative sensitivities defined with eq 1 provide a convenient method of comparing nanothermometers with different architectures and readout modes. However, for practical applications, the relevant parameters are the accuracy and precision of the temperature measurement.^1,11,49^ Accuracy depends on how reliable the calibration procedure is for a target measurement, which usually involves a different nanothermometer environment and experimental setup. Precision on the other hand can be related to properties of the nanoemitters themselves.^49^ The error of the temperature measurement based on reading out *Q*_ξ_ can be written as^1^
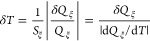
2where δ*Q*_ξ_ is the error in the measurement of *Q*_ξ_. The magnitude of δ*Q*_ξ_ depends primarily on the signal-to-noise ratio in the fluorescence measurement. It can be lowered by using brighter emitters, longer acquisition times, setups with a higher light collection efficiency, or less noisy and more sensitive detectors.^49^ Ultimately, measurements with higher counts will provide a higher precision of the temperature readout. Considering the readout modes based on *Q*_I_ and *Q*_τ_, eq 2 indicates that achieving low readout error δ*T* is conditional on two opposing requirements. On the one hand, δ*T* decreases with the recorded PL intensity. Therefore, one may expect a lower error for emitters with a higher PL QY. However, as the results shown in Figure 3A and C suggest, QDs with a higher PL QY exhibit a lower sensitivity, which will lead to an increased error. Clearly, to optimize the performance of QD-based nanothermometers, balancing the PL QY and sensitivity will be the key task.

### Multiparametric Readout Based on MLR

Having readout modes based on independent spectroscopic observables allows combining them into a multiparametric readout using MLR. In essence, the approach relies on constructing a function
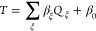
3and finding the parameters β_ξ_ and β_0_ by performing MLR. Note that β values are weighted inverse slopes of the linear functions *Q*_ξ_(*T*) or, in other words, weighted inverse absolute sensitivities. As we show below, the benefit of the MLR approach is a significant increase in the thermometer sensitivity and a decrease in the readout error. Since the estimation of the response variable, temperature, is based on four predictor variables, *Q*_ξ_, the sensitivity of the multiparametric readout is given by (cf. eq 1 and ref (39))
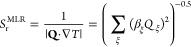
4In the above equation, **Q** is a vector in the space of the predictor variables and ∇*T* is a gradient of the calibration function given by eq 3. The readout error in the MLR mode reads (cf. eq 2)
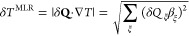
5where δ**Q** is a vector containing the measurement errors δ*Q*_ξ_.

We perform fitting of the MLR model to the data for all the samples. (For more information on the procedure, see the Supporting Information, Section S7, equations S1 and S2, and Figure S13.) The sensitivities of the MLR readout mode for all of the samples are marked in Figure 3E as red stars. Clearly, *S*_r_^MLR^ values are significantly larger than those for sensitivities of single-parameter readout modes. For CIS/ZnS-30, we obtain *S*_r_^MLR^ = 23% °C^–1^, a value which is more than 7 times larger than *S*_I_, which is the highest value of all single-parameter sensitivities. For all samples, *S*_r_^MLR^ exceeds 5% °C^–1^ and exceeds maximum single-parameter readout sensitivities by a factor of 5 on average. Crucially, the data in Figure 3E show that our multiparametric approach makes CIS/ZnS QDs the most sensitive nanothermometers among semiconducting QD systems emitting in the first biological window.

**Figure 4:**
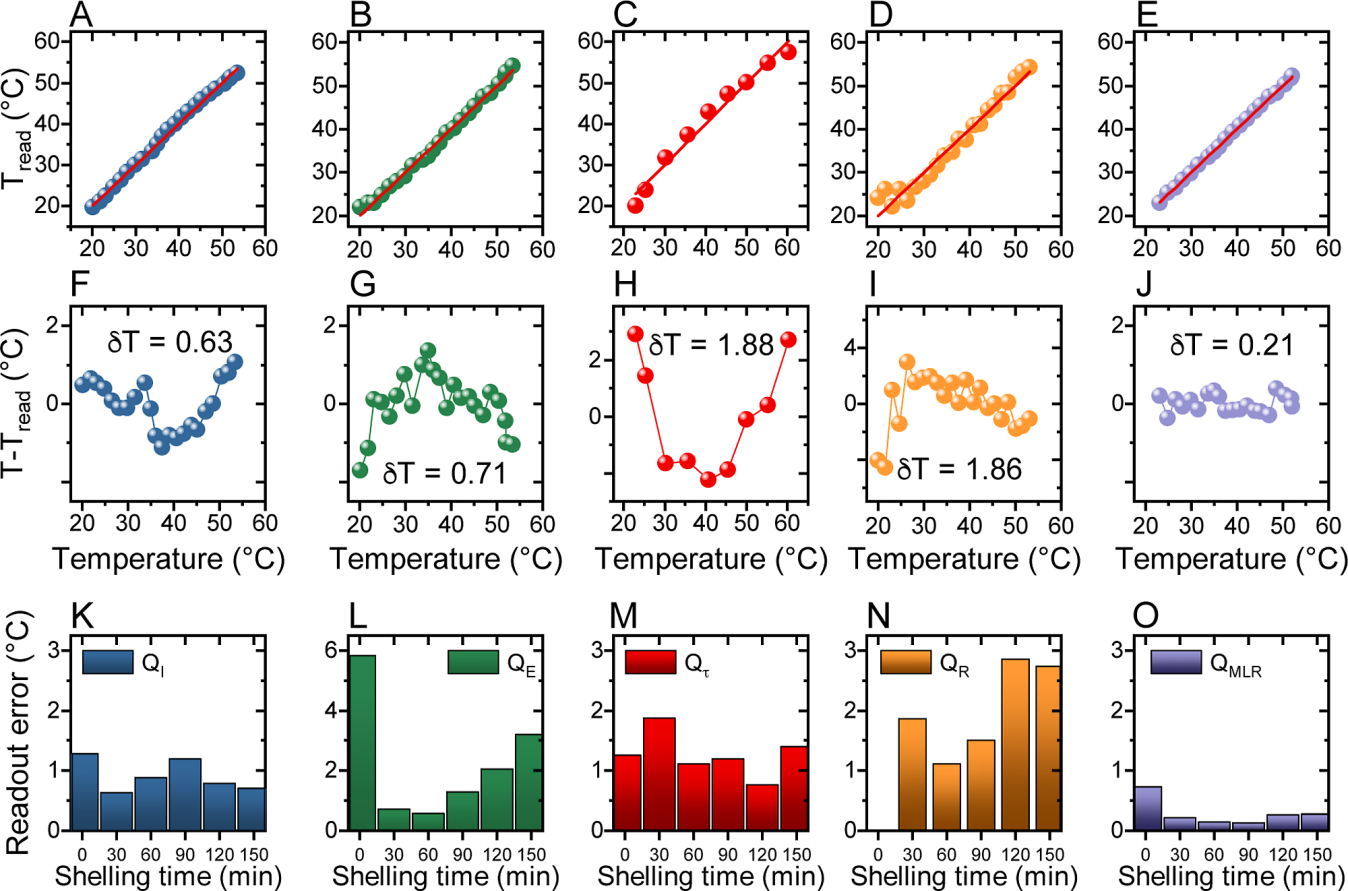
(A–E) Readout temperature *T*_read_ based on PL intensity, energy, lifetime, excitation ratio, and MLR model, respectively, plotted as a function of the sample temperature. Lines denote *T*_read_ = *T*. (F–J) Dependence of *T*–*T*_read_ residuals on the sample temperature. Values given in the graphs indicate the readout error defined as a standard deviation of the distribution of *T*–*T*_read_ residuals. (K–O) Readout errors evaluated for all samples for the modes based on PL intensity, energy, lifetime, excitation ratio, and MLR model, respectively.

To demonstrate the impact of the multiparametric readout on accuracy and readout error, we read out temperatures from the calibration data sets. Namely, we apply the calibration functions *T*(*Q*_ξ_) to the measured temperature-dependent PL intensity, PL energy, lifetime, and excitation ratio. This allows comparison of the readout temperature *T*_read_ with the sample temperature *T* (i.e., the temperature at which the PL properties were recorded) and evaluation of the readout error. The results for sample CIS/ZnS-30 are plotted in Figure 4A–D for measurements of *Q*_*I*_, *Q*_E_, *Q*_τ_, and *Q*_R_, respectively. In Figure 4E, we plot *T*_read_ vs *T* for the MLR readout. In Figure 4F–J, we plot the residuals *T*–*T*_read_. We evaluated the readout error δ*T* as the standard deviation of the distribution of these residuals. The obtained δ*T* values are shown in the corresponding graphs. These results clearly indicate that the MLR readout affords the smallest readout error, decreased by a factor of 3 with respect to the smallest error of the single-parameter mode (*Q*_I_ in the case of this sample). Readout errors established in this way for all the samples are shown in Figure 4K–O. The results clearly indicate that the MLR readout greatly decreases the error for all samples.

## Conclusions

In conclusion, we demonstrated that multiparametric temperature readout afforded by core/shell CIS/ZnS colloidal QDs makes them the most sensitive nanothermometers in the family of semiconducting QDs. The record sensitivities are obtained by combining, via MLR, four single-parameter readout modes based on measurements of PL intensity, peak energy, lifetime, and a specific excitation ratio. While the sensitivities of the single-parameter readout modes are on par with other semiconducting QD systems, the MLR mode boosts the sensitivity by a factor of 8. Furthermore, we showed that the readout error in the multiparametric mode is more than 3 times smaller than for single-parameter readouts. We investigated the thermometer performance for a series of samples with different shelling times and discussed the influence of the PL QY on sensitivity and error. Our study was performed on hydrophilic QDs boasting good stability in aqueous solution and with a more environmentally friendly composition compared to the commonly investigated Pb and Cd chalcogenides. Therefore, our results indicate that CIS/ZnS QDs are perfectly suited for demanding, high sensitivity in vivo intracellular thermometry. In general terms, our study demonstrates a route for achieving better performance with other nanothermometer materials by combining single-parameter readouts into a multiparametric one.

## Materials and Methods

### Materials

All chemicals are commercially available and were used without further purification: copper(I) iodide (CuI, 99.9%, Sigma-Aldrich), indium acetate (In(Ac)_3_, 99.9%, Sigma-Aldrich), DDT (98%, Sigma-Aldrich), toluene (pure p.a., Chempur), acetone (99.5%, Avantor Performance Materials Poland S.A.), zinc stearate (technical grade, Sigma-Aldrich), OA, 1-octadecene (ODE, technical grade, 90%, Sigma-Aldrich), chloroform, polyoxyethylene (40) stearate (PS, Sigma-Aldrich), and ethanol (99.9%, Sigma-Aldrich).

### Synthesis of CuInS_2_ QDs

CIS QDs were synthesized according to the previously reported procedure from refs (29 and 41). Into a three-necked flask, 0.292 g of indium acetate, 0.190 g of copper(I) iodide, and 5 mL of DDT were added. The reaction mixture was degassed at room temperature under an argon flow (0.5 L/min). After 30 min, the temperature was raised to 100 °C. When the solution became clear and colorless, the temperature was raised to 230 °C. After 10 min, the synthesis was ended by rapidly cooling the mixture in a water bath. The QDs were precipitated with an excess of acetone. Then, the samples were centrifuged for 10 min (6000 rpm, 15 °C), and the precipitate was decanted. The supernatant was redispersed in toluene, and the process was repeated 3 times. The final product was dispersed in toluene.

### Shelling of CuInS_2_ QDs with ZnS

CIS cores were overgrown with ZnS shells as described in the procedure given by Speranskaya et al. in ref (42). To prepare the zinc precursor, 1.6 g of zinc stearate, 14 mL of ODE, 4.3 mL of OA, and 0.84 mL of DDT were added to the three-neck flask. The reagents were mixed and degassed under argon flow (0.5 L/min). Next, the temperature of the reaction mixture was raised to 190 °C. When the solution became clear and homogeneous, the temperature was decreased to 75 °C. Simultaneously, CIS QDs synthesized using the procedure above were cooled to 80 °C. Next, 2.7 mL of prepared zinc precursor was added to the QDs, and the temperature was raised to 215 °C. After 30 min, the solution was cooled to 80 °C, and a small aliquot was taken. Then, another 2.7 mL volume of Zn precursor was added, and the temperature was raised again to 215 °C. Overall, 5 such cycles were performed, and an aliquot was taken after each cycle. After the synthesis, the core–shell QDs were precipitated and washed using the same procedure as for core-only QDs. The final product was dispersed in toluene.

### Encapsulation in Micelles

CIS and CIS/ZnS QDs were encapsulated in micelles following the method described in ref (36). The QDs (1 mg) dispersed in chloroform, 50 mg of PS, and 2 mL of chloroform were added to a round-bottom flask. When PS was dissolved, chloroform was evaporated from the reaction mixture. After obtaining a thin film of QDs with PS, 5 mL of water was added, and the temperature was raised to 70 °C. Next, the solution was sonicated. Heating and sonication of the obtained solution were repeated until a uniform solution was obtained. The final product was filtered through a 0.2 μm membrane filter to remove aggregates. QDs encapsulated in micelles were dispersed in distilled water.

### Structural Characterization

For the studies of the QD morphology, the samples were diluted to micromolar concentrations. QDs were imaged with a TALOS F200X transmission electron microscope operating at 200 kV.

### Optical Measurements

Absorbance measurements were performed on a Varian Cary 50 UV–vis spectrophotometer. QY measurements were performed on a Cary Eclipse fluorescence spectrophotometer. The procedure for QY evaluation is given in Supporting Information, Section S2. Temperature-dependent PL spectra were measured with a HORIBA Jobin Yvon Fluorolog-3 spectrofluorometer. The sample in a quartz cuvette was placed in a custom-built holder, allowing for varying and monitoring the temperature with ethylene glycol as the heat exchange liquid. The PL spectra were corrected for the detector sensitivity. The PL decay times were measured by using a custom-built setup. A quartz cuvette containing an aqueous suspension of QDs was immersed in a water-containing dish and placed on a heating plate. The temperature was controlled by using a thermocouple immersed in the cuvette. A pulsed laser emitting at 400 nm was used as the excitation source at a 50 kHz repetition rate. The signal was collected in the direction perpendicular to the excitation beam. Scattered laser light was rejected with a long-pass filter. The signal was focused onto an avalanche photodiode (Excelitas SPCM-AQRH-15) and the time-resolved signal was histogrammed by a time-correlated single photon counting unit (PicoQuant PicoHarp 300). The overall temporal resolution was ∼1 ns.

## Supporting Information Available

Transmission electron microscopy image of CIS/ZnS-30 QDs; optical characterization (absorbance spectra, first excitonic transition, and PL spectra) of CIS/ZnS QDs series (ZnS shelling time from 0 to 150 min); PL QY of the CIS/ZnS QD series; comparison of PL spectra measured for samples in toluene and, after encapsulation, in water; results of stability and reversibility studies on sample CIS/ZnS-30; temperature dependence of PL intensity, PL peak shift, and PL lifetime for CIS/ZnS-0, CIS/ZnS-60, and CIS/ZnS-150 QDs; normalized PL spectra measured at 20 and 54 °C; operation of the ratiometric mode for CIS/ZnS-60, CIS/ZnS-90, and CIS/ZnS-150 QDs; table containing excitation wavelengths used in ratiometric temperature readout; tables comparing the temperature sensitivities for different QDs based on PL intensity, PL lifetime, and ratiometric mode; discussion of the mechanism behind the temperature-dependent PL properties; and additional information on the multiple regression analysis of the data (PDF)
