# Supplementary material for: Multimodal Temperature Readout Boosts the Performance of CuInS2/ZnS Quantum Dot Nanothermometers
Source: ACS Appl Mater Interfaces. 2024 Oct 22;16(44):60008–17. doi: 10.1021/acsami.4c14541 (PMC11551904; doi:10.1021/acsami.4c14541)
Supplement: Supplementary file 1 — am4c14541_si_001.pdf [file am4c14541_si_001.pdf]

# Supporting Information for Multimodal Temperature Readout Boosts the Performance of $CuInS_2$/ZnS Quantum Dot Nanothermometers

Magdalena Duda,† Pushkar Joshi,† Anna Borodziuk,† Kamil Sobczak,‡ Bożena Sikora-Dobrowolska,† Sebastian Maćkowski,¶ Allison M. Dennis,§ and Łukasz Kłopotowski*,†

†*Institute of Physics, Polish Academy of Sciences, 02-668 Warsaw, Poland*

‡*University of Warsaw Biological and Chemical Research Centre, 02-089 Warsaw, Poland*

¶*Institute of Physics, Faculty of Physics, Astronomy and Informatics, Nicolaus Copernicus University, 87-100 Toruń, Poland*

§*Department of Chemical Engineering, Northeastern University, Boston, MA 02115, United States*

E-mail: lukasz.klopotowski@ifpan.edu.pl

# Contents


**S1 Structural characterization** **S3**

**S2 Optical characterization** **S3**

**S3 Stability and reversibility studies** **S5**

**S4 Temperature-dependent optical properties of other samples** **S7**

**S5 Origin of temperature-dependent optical properties** **S13**

**S6 Comparison of nanothermometer sensitivities** **S16**

**S7 Notes on Multiple Regression Analysis** **S18**

**References** **S20**

# S1 Structural characterization

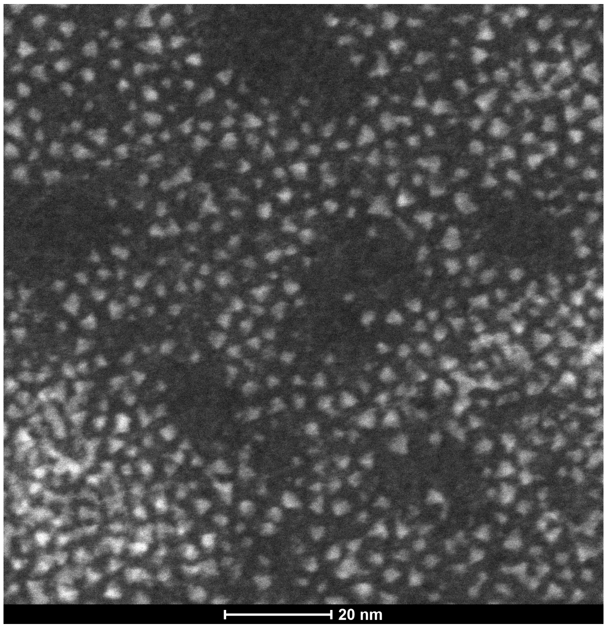


Figure S1: STEM-HAADF image of CIS/ZnS-30. The scale bar corresponds to 20 nm. The image reveals pyramid-shaped nanocrystals with an average edge length of about 3 nm.

# S2 Optical characterization

The QY of the prepared samples was determined using the relative approach to the QY of the known fluorescent standard. The PL QY standard used was Rhodamine 6G dispersed in ethanol. The excitation wavelength was set at 488 nm. The QY was calculated based on equation:[1]

$$QY_x = QY_{st}\frac{F_x}{F_{st}}\frac{f_{st}}{f_x}\frac{n_x^2(\lambda_{em})}{n_s^2(\lambda_{em})}$$

where $QY_{st}$ is equal to 95%, $f_{st}$ and $f_x$ are the absorption factors of the sample and standard respectively, $F_{st}$ and $F_x$ are the PL integrals of the standard and the sample respectively, and n stands for the refractive indexes of the used solvents.

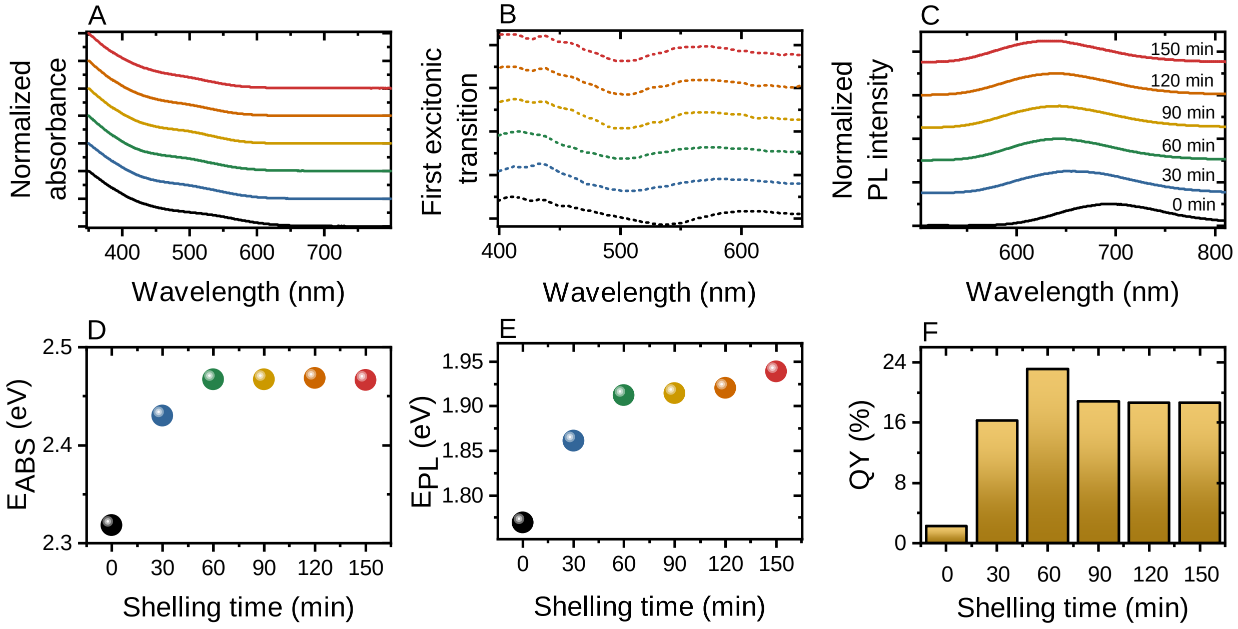


Figure S2: A: Absorption spectra (solid lines) B: The first excitonic transition (dotted lines) and C: PL spectra for CIS/ZnS QDs with different ZnS synthesis times. D: CIS/ZnS QDs first excitonic transition as a function of ZnS shelling time. E: CIS/ZnS QDs PL maximum as a function of ZnS shelling time. F: CIS/ZnS QDs PL QY as a function of ZnS shelling time.

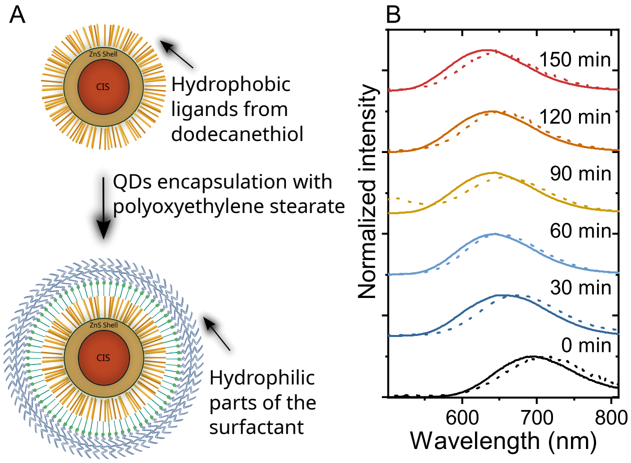


Figure S3: A: Scheme of CIS/ZnS QDs encapsulation in micelles. B: PL spectra for CIS/ZnS QDs dispersed in toluene (solid lines) and CIS/ZnS QDs encapsulated in micelles and dispersed in water (dashed lines).

## S3 Stability and reversibility studies

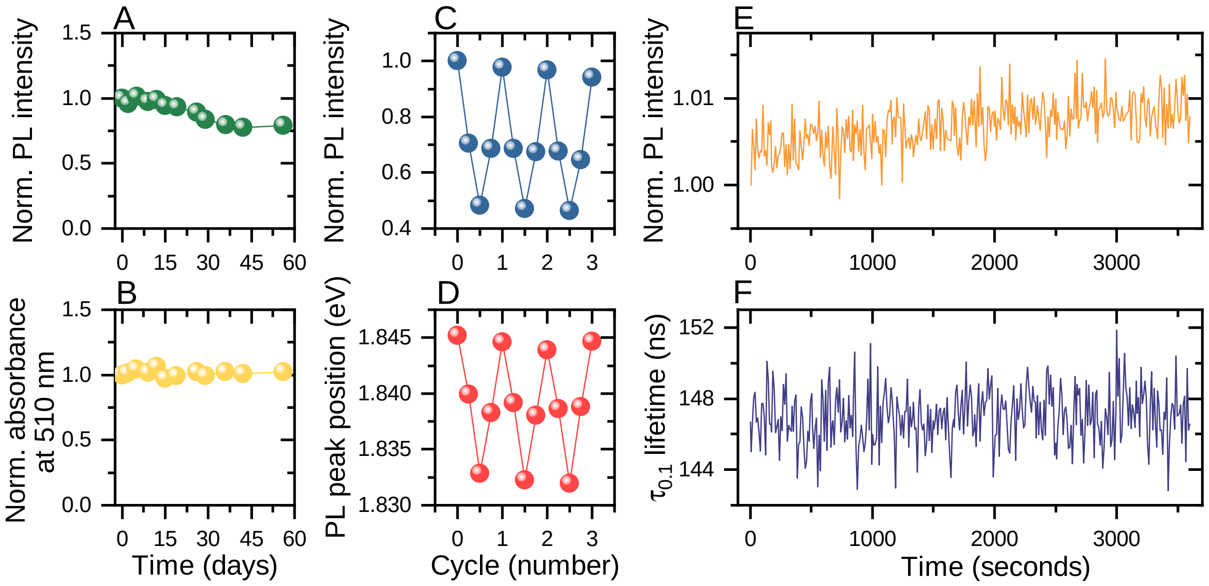


Figure S4: A–B: Stability of the micelle-encapsulated CIS/ZnS-30 QDs dispersed in water studied over 56 days. A: Time dependence of PL intensity excited at 450 nm. B: Time dependence of normalized absorbance at 510 nm. C–D: Thermal stability of the CIS/ZnS-30 QDs. C: PL intensity and D: position of the PL peak measured over three heating and cooling cycles. E–F: Stability of the PL intensity and PL lifetime of the CIS/ZnS-30 QDs under 400 nm excitation.

To investigate long term stability of micelle-encapsulated CIS/ZnS QDs in aqueous solutions, we monitored absorbance and PL over 56 days. The sample was kept in the cuvette throughout the experiment and was refrigerated between measurements. To ensure reproducible experimental conditions, the cuvette was weighed before each measurement to verify concentration constancy. Before the measurements, the sample temperature was stabilized for 15 minutes. In Figure S4A and B we present, respectively, the integrated PL intensity under 450 nm excitation and the absorbance at the first excitonic transition (at 510 nm) as a function of time. The integrated PL intensity and absorbance were normalized to those obtained on the first day of measurements.

To demonstrate the reversibility of the temperature-dependent processes providing the first two thermometer readout modes, we performed PL measurements upon repeated heating

and cooling. The results are shown in Figs. S4C and D. We find that during 3 heating/cooling cycles, an experiment that lasted 2 hours, the overall decrease of PL intensity was smaller than 5%. At the same time, the PL spectrum returned to a position within 1.1 meV of the initial energy value. Similar results were obtained for the remaining samples.

For practical applications, it is important that the optical properties remain stable over the timescale of an experiment in which the QDs are subject to laser excitation. In Fig. S4E and F, we plot the normalized PL intensity and the PL lifetime measured under continuous excitation with a 400 nm laser. The photons were detected with an avalanche photodiode and a time-correlated single photon counting unit registered photon arrival times over the course of an hour. The photons were then counted in 10.5 s time bins. The number of photons (the PL intensity) is plotted as a function of time in Fig. S4E. A small increase by about 1% over the time of an hour is observed. We attribute this to a small decrease of the ambient temperature. Using the thermometer calibration, the intensity increase indicates a decrease of the ambient temperature by 0.3°C. The arrival times of the photons counted in the time bins were then used to construct PL decay profiles, from which the $\tau_{0.1}$ lifetimes were evaluated. The temperature dependence of the $\tau_{0.1}$ lifetime is shown in Fig. S4F. Note that the effect of decrease in ambient temperature determined from the temporal dependence of PL intensities (Fig. S4E is not seen here due to large fluctuations in the evaluated $\tau_{0.1}$.

## S4 Temperature-dependent optical properties of other samples

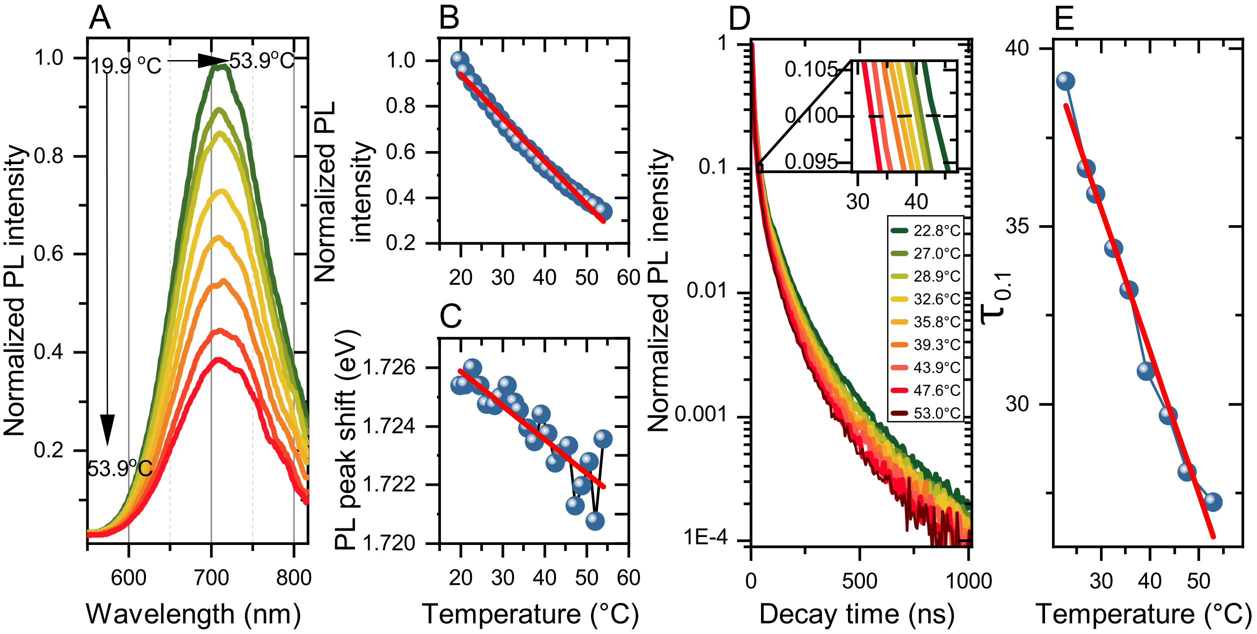


Figure S5: A: PL spectrum measured as a function of temperature for CIS/ZnS-0 encapsulated in micelles. The equation of the fitted linear function is $Q_I(T) = -0.019T + 1.32$. B: Normalized PL intensity as a function of temperature. C: Shift of the PL peak as a function of temperature. The equation of the fitted linear function is $Q_E(T) = -0.000116T + 1.73$. The excitation wavelength was 450 nm. D: Normalized PL decay measured as a function of temperature. E: Temperature dependence of PL lifetime (blue points). The lifetime is defined as the decay time at which the intensity drops by a factor of 10 – see the inset in D. The lifetimes are fitted with a linear function $Q_\tau(T) = -0.4T + 47$ (red line).

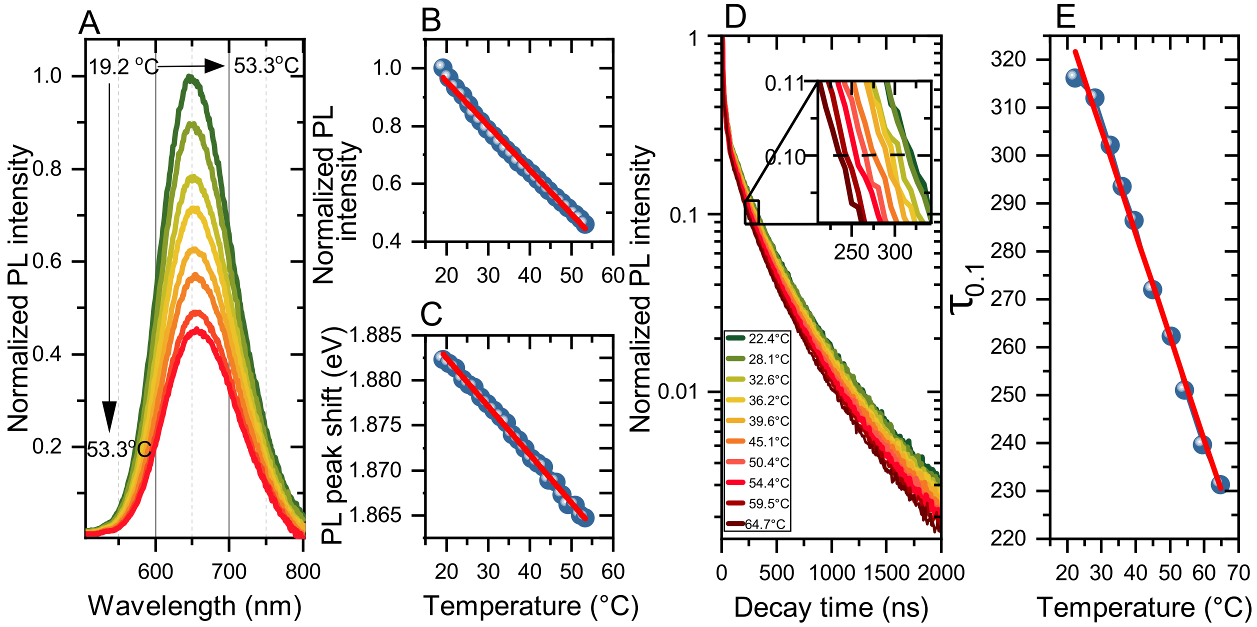


Figure S6: A: PL spectrum measured as a function of temperature for CIS/ZnS-60 encapsulated in micelles. The equation of the fitted linear function is $Q_I(T) = -0.0153T + 1.26$. B: Normalized PL intensity as a function of temperature. C: Shift of the PL peak as a function of temperature. The equation of the fitted linear function is $Q_E(T) = -0.00054T + 1.89$. The excitation wavelength was 450 nm. D: Normalized PL decay measured as a function of temperature. E: Temperature dependence of PL lifetime (blue points). The lifetime is defined as the decay time at which the intensity drops by a factor of 10 – see the inset in D. The lifetimes are fitted with a linear function $Q_\tau(T) = -2.15T + 370$ (red line).

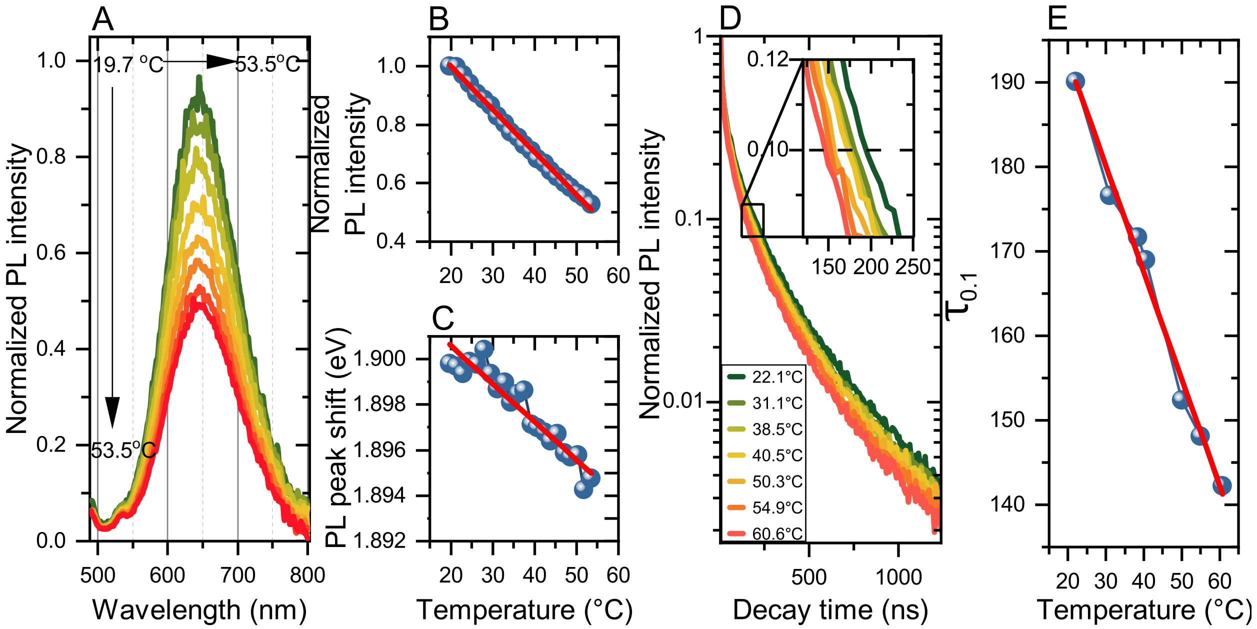


Figure S7: A: PL spectrum measured as a function of temperature for CIS/ZnS-150 encapsulated in micelles. The equation of the fitted linear function is $Q_I(T) = -0.0146T + 1.29$. B: Normalized PL intensity as a function of temperature. C: Shift of the PL peak as a function of temperature. The excitation wavelength was 450 nm. The equation of the fitted linear function is $Q_E(T) = -0.000168T + 1.9$. D: Normalized PL decay measured as a function of temperature. E: Temperature dependence of PL lifetime (blue points). The lifetime is defined as the decay time at which the intensity drops by a factor of 10 – see the inset in D. The lifetimes are fitted with a linear function $Q_\tau(T) = -1.26T + 218$ (red line).

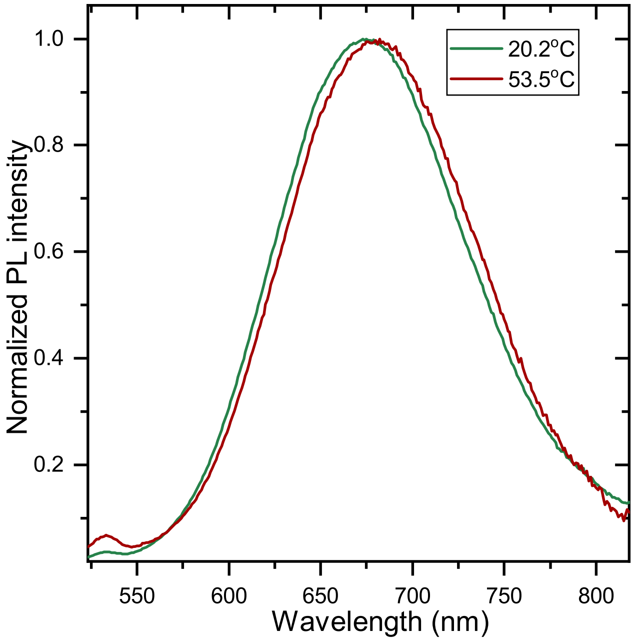


Figure S8: Normalized PL intensity measured at 20.2°C and 53.5°C for CIS/ZnS-30 encapsulated in micelles and dispersed in water.

Table S1: Values of first and second excitation wavelength used for dual-wavelength excitation in ratiometric temperature readout for CIS/ZnS samples with ZnS shelling time varying from 30 to 150 min.

| Sample name | CIS/ZnS-30 | CIS/ZnS-60 | CIS/ZnS-90 | CIS/ZnS-120 | CIS/ZnS-150 |
|---|---|---|---|---|---|
| I excitation wavelength | 450 nm | 450 nm | 450 nm | 450 nm | 450 nm |
| II excitation wavelength | 580 nm | 576 nm | 568 nm | 566 nm | 563 nm |

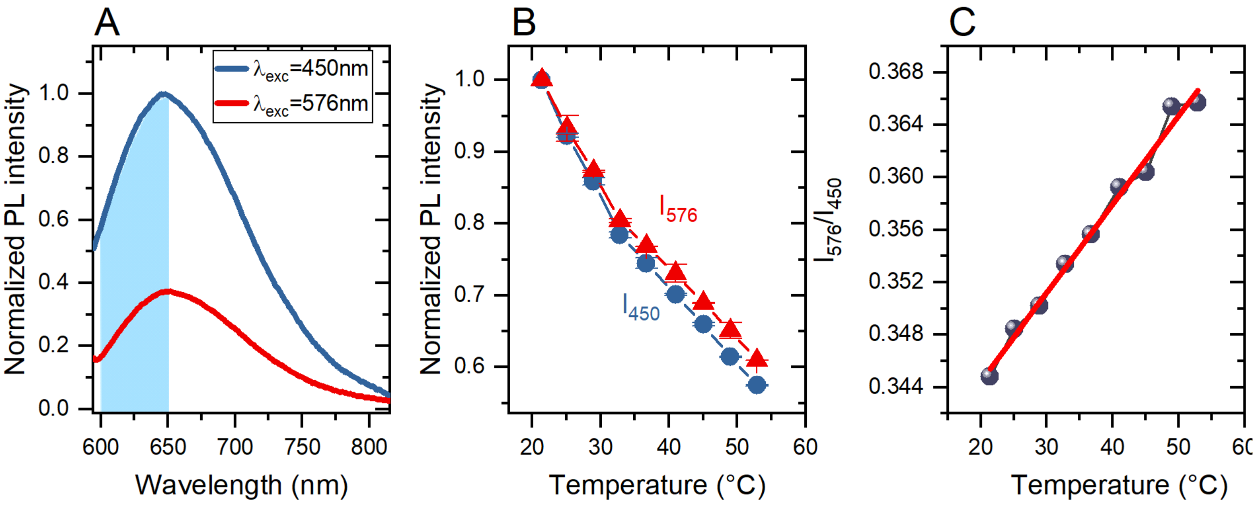


Figure S9: A: PL spectra after 450 (blue line) and 576 nm (red line) excitation for CIS/ZnS-60 encapsulated in micelles and dispersed in water. The highlighted area denotes the spectrum integration range. B: Influence of different excitation wavelengths on PL intensity as a function of temperature. Excitation wavelengths were 450 (blue dots) and 576 nm (red triangles) C: Temperature dependence of $I_{450}/I_{576}$ intensity ratio (blue points) and a fitted linear function is $Q_R(T) = 0.00068T + 0.33$ (red line).

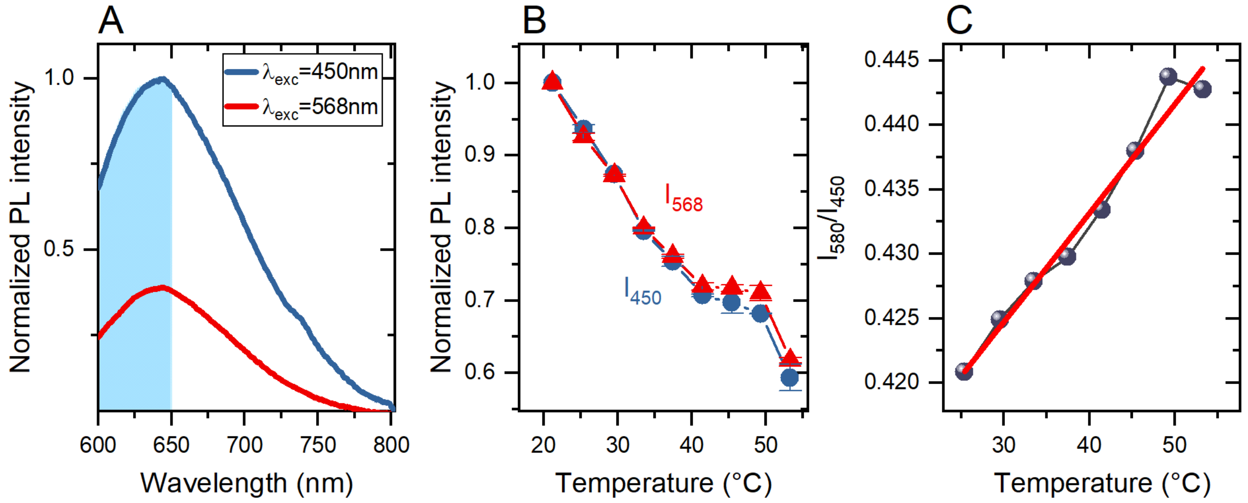


Figure S10: A: PL spectra after 450 (blue line) and 568 nm (red line) excitation for CIS/ZnS-90 encapsulated in micelles and dispersed in water. The highlighted area denotes the spectrum integration range. B: Influence of different excitation wavelengths on PL intensity as a function of temperature. Excitation wavelengths were 450 (blue dots) and 568 nm (red triangles) C: Temperature dependence of $I_{450}/I_{568}$ intensity ratio (blue points) and a fitted linear function is $Q_R(T) = 0.00084T + 0.399$ (red line).

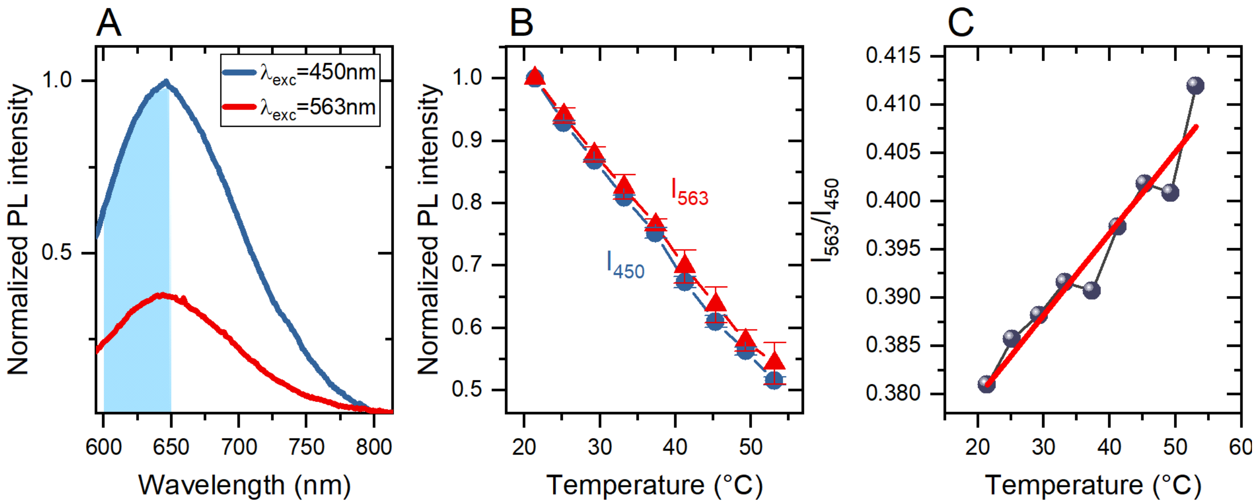


Figure S11: A: The PL spectra after 450 (blue line) and 563 nm (red line) excitation for CIS/ZnS-150 encapsulated in micelles and dispersed in water. The highlighted area denotes the spectrum integration range. B: Influence of different excitation wavelengths on PL intensity as a function of temperature. Excitation wavelengths were 450 (blue dots) and 563 nm (red triangles). C: Temperature dependence of $I_{450}/I_{563}$ intensity ratio (blue points) and a fitted linear function is $Q_R(T) = 0.00085T + 0.36$ (red line).

# S5 Origin of temperature-dependent optical properties

Here, we discuss the physical mechanisms behind the temperature dependent optical properties shown in Figs. 1 and 2 that enable the temperature readouts. Similarly to the case of lead and cadmium chalcogenides, the non-radiative recombination in CIS and CIS/ZnS QDs has been attributed to thermal activation of surface trapping[2–6] In a simple picture, the PL intensity is proportional to PL QY given by $\eta = k_r/(k_r + k_{nr}(T))$, where $k_r$ and $k_{nr}(T)$ are radiative and non-radiative decay rates. The former can be considered as temperature-independent above room temperature, while the latter is thermally activated. The measured PL lifetime is equal to $1/(k_r + k_{nr}(T))$. Therefore, we expect that the decrease of the PL QY and the shortening of the PL lifetime occur with the same rate. However, the PL intensity upon excitation at 400 nm decreases by about 45% (see Supporting Information Fig. S11), while in the same temperature range $\tau_{0.1}$ decreases by only 32% (Fig. 1E). This discrepancy shows that there exists an additional process, which drives the thermal quenching of PL intensity, while not affecting the PL lifetimes.

To explain in detail the mechanism behind PL quenching and shortening of the PL lifetime, we recall that surface trapping can be described as an electron transfer process between a QD state and a coupled surface state.[7,8] This process is schematically depicted in Fig. S12 in a configuration coordinate diagram showing the energies of the QD ground and excited states as well as the surface trap state. Following photoexcitation with a high excess energy the system is transferred from the ground state, at point A, to the excited state at point B'. From B' the system can undergo an energy relaxation within core states toward the emissive state at point C. From C, there are also two possible pathways: radiative recombination via a transition to point D or surface trapping, i.e., transfer to point G. Relaxation from point G to the ground state at point H is a non-radiative process. The trapping requires surmounting a barrier given by the energy difference between points C and

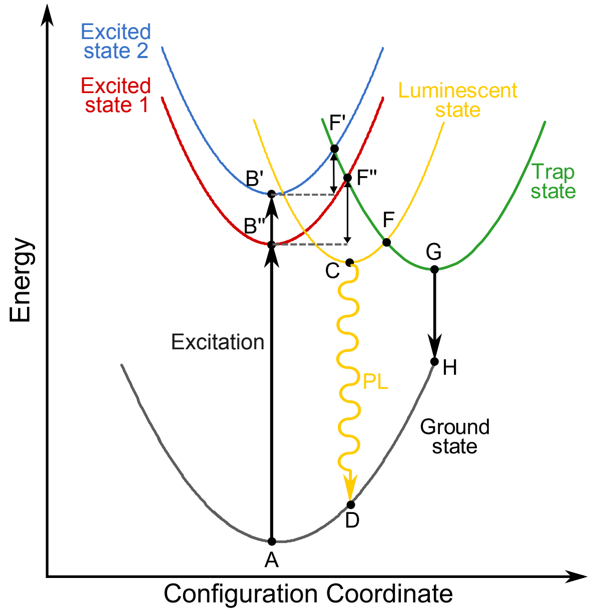


Figure S12: Configuration coordinate diagram depicting the excitation, trapping, and recombination processes responsible for temperature dependent PL processes. Solid upward and downward arrows denote, respectively, excitation and non-radiative recombination. Wavy arrow denotes radiative recombination. Double headed arrows show the barrier heights for hot carrier trapping. See text for details.

F, $\Delta_{CF}$. Thus, non-radiative recombination via surface trapping is a thermally activated process, which results in a shortening of the observed PL lifetime and a concomitant loss of PL QY. Since both of these effects are driven by the same surface trapping process, their rates are the same as expected from the simple picture discussed in the previous paragraph. However, from point B' a second energy relaxation pathway is available: transfer to point G via a barrier between points B' and F', $\Delta_{B'F'}$. This is a hot carrier trapping effect that reduces the PL QY, by removing part of the carriers before relaxation to the emissive state, i.e., without influencing the observed PL lifetime.[9] The discussion above allows us to attribute the decrease of the PL intensity to the combined effect of thermally activated hot (from B' to G via F') and cold carrier trapping (from C to G via F). On the other hand, the shortening of the PL lifetimes is a consequence of cold carrier trapping alone. The distinction between hot and cold carrier trapping thus explains the discrepancy between the PL intensity quenching and PL lifetime shortening rates observed in Fig. 1 and reported in

Ref. 3.

Notably, the configuration coordinate diagram presented in Fig. S12 suggests a mechanism behind the ratiometric temperature readout mode. Let us compare the scenarios for excitations with low and high excess energy, namely reaching, respectively, points B" and B' upon photoexcitation. In these two cases, we find that the barriers for carrier trapping are different: $\Delta_{B''F''} > \Delta_{B'F'}$. Thus, excitation with a smaller excess energy creates carriers, which experience a higher energy barrier for trapping route B" to G via F" than excitation with a higher excess energy (B' to G via F'). As a result, surface trapping following excitation with a lower energy has a smaller efficiency leading to an increased PL QY compared to the case of a higher excitation energy. Because of the difference in barrier heights, thermally induced PL quenching for lower excitation energy is weaker than for higher excitation energy as indeed observed in the experimental data presented in Fig. 2B. Therefore, the discussion of the configuration coordinate diagram allows us to attribute the fourth mode of the temperature readout to different rates of hot and less hot electron trapping. We note that this difference was shown to underlie the excitation energy dependence of PL QY observed experimentally for CdSe QDs[10] and, very recently, for CIS/ZnSSe core/shell QDs.[11] Finally, we attribute the PL energy redshift with the temperature (Figs. 1C and S6-8C) to the closing of the semiconductor bandgap.

# S6 Comparison of nanothermometer sensitivities

Table S2: Comparison of the QD-based nanothermometer sensitivities operating by measurement of the PL intensity. Rows highlighted in green correspond to systems that emit in the I and II biological window.

| QDs | $\lambda_{em}$ | Size (nm) | Temperature range (°C) | Sensitivity (%/°C) | Ref. |
|---|---|---|---|---|---|
| CdTe | 518 | 4 | 30-60 | 1.1 | 12 |
| CdTe-QDs-LDH | 558 | 2.7 | 23-80 | 1.5 | 13 |
| $ZnS:Mn^{2+}$ | 589 | 10 | 30-150 | 0.5 | 12 |
| CdSe/ZnS | 590 | 4.8-5.5 | -173-42 | 1.3 | 14 |
| CdSe/ZnS | 620 | | 10-80 | 0.7 | 15 |
| $CuInS_2$/ZnS | 647 | 3.9 | 0-60 | 2 (at room T) | 16 |
| $CuInS_2$/ZnS-30 | 682 | | 20-54 | 3.1 | This work |
| PbS/CdS/ZnS | 1270 | 4 | 10-60 | 1 | 17 |

Table S3: Comparison of the QD-based nanothermometer sensitivities operating by measurement of the PL peak shift. Rows highlighted in green correspond to systems that emit in the I and II biological window.

| QDs | $\lambda_{em}$ | Size (nm) | Temperature range (°C) | Sensitivity (nm/°C) | Ref. |
|---|---|---|---|---|---|
| CdTe | 515 | 1.2 | 25-70 | 0.8 | 18 |
| CdSe | 535 | 4.2 | 30-100 | 0.095 | 19 |
| CdTe-QDs-LDH | 558 | 2.7 | 23-80 | 0.193 | 13 |
| $ZnS:Mn^{2+}$ | 589 | 10 | 30-150 | 0.05 | 12 |
| CdSe/ZnS/$SiO_2$ | 606 | 3-4 | 22-252 | 0.11 | 20 |
| $CuInS_2$/ZnS-60 | 653 | | 19-53 | 0.18 | This work |
| CdSe | 655 | 4 | 30-60 | 0.16 | 21 |
| CdTe | 660 | 3.8 | 25-70 | 0.32 | 18 |

Table S4: Comparison of the QD-based nanothermometer sensitivities operating by measurement of the PL lifetimes. Rows highlighted in green correspond to systems that emit in the I and II biological window.

| QDs | $\lambda_{em}$ | Size (nm) | Temperature range (°C) | Sensitivity (%/°C) | Ref. |
|---|---|---|---|---|---|
| CdTe | 510 | 1 | 27-50 | 1.7 | 22 |
| PbS/CdS | 630 | 12 | -123-77 | 1.3 | 23 |
| CdSe | 650 | 4 | 22-48 | 0.08 | 22 |
| PbS/CdS/CdSe | 670 | 9.7 | -173-27 | 1.6 (for CdSe shell) | 24 |
| $CuInS_2$/ZnS-30 | 682 | | 20-54 | 1 | This work |
| $CuInS_2$ | 709 | 2.3 | 20-54 | 1.2 | This work |
| PbS/CdS/CdSe | 910 | 9.7 | -173-27 | 0.5 (for PbS core) | 24 |

Table S5: Comparison of the QD-based nanothermometer sensitivities based on ratiometric mode. Rows highlighted in green color correspond to systems that emit in the I and II biological window.

| QDs | $\lambda_{em}$ | $\lambda_{exc}$ | Size (nm) | Temperature range (°C) | Sensitivity (%/°C) | Ref. |
|---|---|---|---|---|---|---|
| CdSSe/ZnS:$Mn^{2+}$ | $\lambda_1$=520<br>$\lambda_2$=600 | 400 | 10.4 | 20-50 | 0.3 | 25 |
| $Zn_{1-x}Mn_xSe$/ZnS/CdS/ZnS | $\lambda_1$=520<br>$\lambda_2$=600 | | 5 | -140-90 | 0.72 | 26 |
| PbS/CdS | $\lambda_1$=480<br>$\lambda_2$=630 | 400 | 12 | -123-100 | 1.22 | 23 |
| CdS/ZnS:$Mn^{2+}$ | $\lambda_1$=600<br>$\lambda_2$=650 | 403 | 7.8 | -196-107 | 0.5 | 27 |
| $CuInS_2$/ZnS-30 | $\lambda$=682 | $\lambda_1$=450<br>$\lambda_2$=580 | | 20-54 | 0.26 | This work |
| PbS/CdS/CdSe | $\lambda_1$=670<br>$\lambda_2$=910 | 560 | 9.7 | -93-37 | 1.13 | 24 |

## S7 Notes on Multiple Regression Analysis

The first note is concerned with data preparation for MLR. As mentioned in the main text, the readout based on multiple regression analysis (MLR) requires finding parameters $\beta$ for a function given by eq. (3):

$$T = \sum_{\xi} \beta_\xi Q_\xi + \beta_0. \tag{S1}$$

In the above equation, $T$ is the temperature and $Q_\xi$ are the temperature-dependent spectroscopic observables – in our case the PL intensity, peak energy, lifetime, and excitation ratio discussed in the main text. To perform the fitting of the MLR model to the data, $Q_\xi$ values have to be measured at the same temperature set. However, the excitation ratios ($Q_R$) and PL lifetimes ($Q_\tau$) were measured at different values of $T$ than the PL intensity ($Q_I$) and PL peak energy ($Q_E$). To perform MLR, we took $Q_\tau$ and $Q_R$ values interpolated at the temperatures, where $Q_I$ and $Q_E$ were measured. The interpolation was linear and performed in the $T$ range where $Q_I$ and $Q_E$ were measured. Thus, no extrapolation of the data was performed.

Secondly, we note that a more general MLR approach would use standardized predictor variables given by $z_\xi = (Q_\xi - \mu_\xi)/\sigma_\xi$, where $\mu_\xi$ and $\sigma_\xi$ are the mean and standard deviation of $Q_\xi$. Such an approach assures that the predictor distributions exhibit a zero mean and a unity standard deviation. As a result, the magnitudes of the obtained $\beta$-weights ($\tilde{\beta}$, where the tilde indicates parameters obtained by fitting the MLR model to standardized predictor variables $z_\xi$) can be interpreted as carrying information on relative contributions of particular predictor variables to the multi-parametric readout. More specifically, the relative $\beta$-weights computed as

$$\beta^r_\xi = |\tilde{\beta}_\xi| / \sum_\xi |\tilde{\beta}_\xi| \tag{S2}$$

tell us how much variance of the independent parameter $T$ is caused by variance in the standardized predictor $z_\xi$. In order to obtain this information from non-standardized predictors,

we multiply the obtained $\beta_\xi$ by the standard deviation of the corresponding $Q_\xi$: $\tilde{\beta}_\xi = \beta_\xi \sigma_\xi$. The resulting contributions, $\beta$-weights $\tilde{\beta}_\xi$, to the MLR readouts for all the samples are shown in Fig. S13.

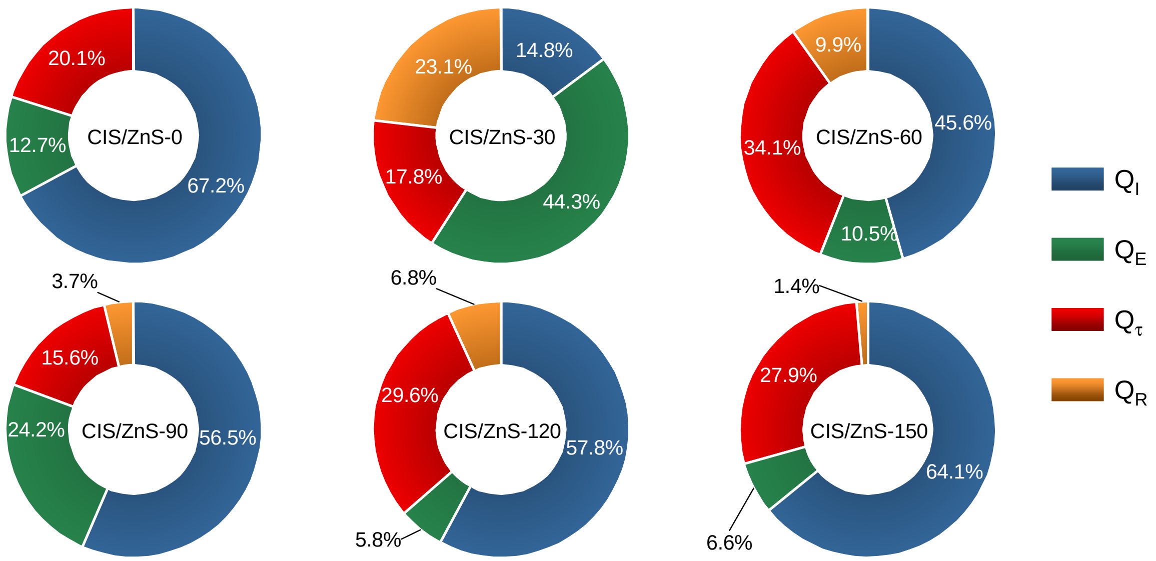


Figure S13: Relative contributions to the MLR readout from single parameter modes based on the measurement of $Q_I$ (blue), $Q_E$ (green), $Q_\tau$ (red) and $Q_R$ (yellow). The values are calculated with eq. S2 and presented for all the shelled samples as indicated.

# References


(1) Würth, C.; Grabolle, M.; Pauli, J.; Spieles, M.; Resch-Genger, U. Relative and Absolute Determination of Fluorescence Quantum Yields of Transparent Samples. *Nature protocols* **2013**, *8*, 1535–1550.

(2) Gaponenko, M. S.; Lutich, A. A.; Tolstik, N. A.; Onushchenko, A. A.; Malyarevich, A. M.; Petrov, E. P.; Yumashev, K. V. Temperature-Dependent Photoluminescence of PbS Quantum Dots in Glass: Evidence of Exciton State Splitting and Carrier Trapping. *Physical Review B* **2010**, *82*, 125320.

(3) Zhao, Y.; Riemersma, C.; Pietra, F.; Koole, R.; de Mello Donegá, C.; Meijerink, A. High-Temperature Luminescence Quenching of Colloidal Quantum Dots. *ACS nano* **2012**, *6*, 9058–9067.

(4) Berends, A. C.; Rabouw, F. T.; Spoor, F. C.; Bladt, E.; Grozema, F. C.; Houtepen, A. J.; Siebbeles, L. D.; de Mello Donegá Celso, Radiative and Nonradiative Recombination in $CuInS_2$ Nanocrystals and $CuInS_2$-Based Core/Shell Nanocrystals. *The journal of physical chemistry letters* **2016**, *7*, 3503–3509.

(5) Sun, J.; Ikezawa, M.; Wang, X.; Jing, P.; Li, H.; Zhao, J.; Masumoto, Y. Photocarrier Recombination Dynamics in Ternary Chalcogenide $CuInS_2$ Quantum Dots. *Physical Chemistry Chemical Physics* **2015**, *17*, 11981–11989.

(6) Szymura, M.; Duda, M.; Karpińska, M.; Kazimierczuk, T.; Minikayev, R.; Sobczak, K.; Parlinńska-Wojtan, M.; Kłopotowski, Ł. Low-Temperature Photoluminescence Dynamics Reveal the Mechanism of Light Emission by Colloidal $CuInS_2$ Quantum Dots. *The Journal of Physical Chemistry C* **2023**, *127*, 6768–6776.

(7) Jones, M.; Lo, S. S.; Scholes, G. D. Quantitative Modeling of the Role of Surface Traps in CdSe/CdS/ZnS Nanocrystal Photoluminescence Decay Dynamics. *Proceedings of the National Academy of Sciences* **2009**, *106*, 3011–3016.

(8) Mooney, J.; Krause, M. M.; Saari, J. I.; Kambhampati, P. A Microscopic Picture of Surface Charge Trapping in Semiconductor Nanocrystals. *The Journal of Chemical Physics* **2013**, *138*, 204705.

(9) Galland, C.; Ghosh, Y.; Steinbrück, A.; Sykora, M.; Hollingsworth, J. A.; Klimov, V. I.; Htoon, H. Two Types of Luminescence Blinking Revealed by Spectroelectrochemistry of Single Quantum Dots. *Nature* **2011**, *479*, 203–207.

(10) Hoy, J.; Morrison, P. J.; Steinberg, L. K.; Buhro, W. E.; Loomis, R. A. Excitation Energy Dependence of the Photoluminescence Quantum Yields of Core and Core/Shell Quantum Dots. *The Journal of Physical Chemistry Letters* **2013**, *4*, 2053–2060.

(11) Ghosh, S.; Mukherjee, S.; Mandal, S.; De, C. K.; Mardanya, S.; Saha, A.; Mandal, P. K. Beneficial Intrinsic Hole Trapping and Its Amplitude Variation in a Highly Photoluminescent Toxic-Metal-Free Quantum Dot. *The Journal of Physical Chemistry Letters* **2023**, *14*, 260–266.

(12) Wang, S.; Westcott, S.; Chen, W. Nanoparticle Luminescence Thermometry. *The Journal of Physical Chemistry B* **2002**, *106*, 11203–11209.

(13) Liang, R.; Tian, R.; Shi, W.; Liu, Z.; Yan, D.; Wei, M.; Evans, D. G.; Duan, X. A Temperature Sensor Based on CdTe Quantum Dots–Layered Double Hydroxide Ultrathin Films via Layer-By-Layer Assembly. *Chemical Communications* **2013**, *49*, 969–971.

(14) Walker, G. W.; Sundar, V. C.; Rudzinski, C. M.; Wun, A. W.; Bawendi, M. G.; Nocera, D. G. Quantum-Dot Optical Temperature Probes. *Applied Physics Letters* **2003**, *83*, 3555–3557.

(15) Han, B.; Hanson, W. L.; Bensalah, K.; Tuncel, A.; Stern, J. M.; Cadeddu, J. A. Development of Quantum Dot-Mediated Fluorescence Thermometry for Thermal Therapies. *Annals of biomedical engineering* **2009**, *37*, 1230–1239.

(16) Zhang, H.; Wu, Y.; Gan, Z.; Yang, Y.; Liu, Y.; Tang, P.; Wu, D. Accurate Intracellular and in Vivo Temperature Sensing Based on $CuInS_2$/ZnS QD Micelles. *Journal of Materials Chemistry B* **2019**, *7*, 2835–2844.

(17) del Rosal, B.; Carrasco, E.; Ren, F.; Benayas, A.; Vetrone, F.; Sanz-Rodríguez, F.; Ma, D.; Juarranz, Á.; Jaque, D. Infrared-Emitting QDs for Thermal Therapy with Real-Time Subcutaneous Temperature Feedback. *Advanced Functional Materials* **2016**, *26*, 6060–6068.

(18) Maestro, L. M.; Jacinto, C.; Silva, U. R.; Vetrone, F.; Capobianco, J. A.; Jaque, D.; Solé, J. G. CdTe Quantum Dots as Nanothermometers: Towards Highly Sensitive Thermal Imaging. *small* **2011**, *7*, 1774–1778.

(19) Sung, T.-W.; Lo, Y.-L. Dual Sensing of Temperature and Oxygen Using PtTFPP-Doped CdSe/$SiO_2$ Core–Shell Nanoparticles. *Sensors and Actuators B: Chemical* **2012**, *173*, 406–413.

(20) Pugh-Thomas, D.; Walsh, B. M.; Gupta, M. C. CdSe (ZnS) Nanocomposite Luminescent High Temperature Sensor. *Nanotechnology* **2011**, *22*, 185503.

(21) Maestro, L. M.; Rodríguez, E. M.; Rodríguez, F. S.; la Cruz, M. I.-d.; Juarranz, A.; Naccache, R.; Vetrone, F.; Jaque, D.; Capobianco, J. A.; Solé, J. G. CdSe Quantum Dots for Two-Photon Fluorescence Thermal Imaging. *Nano letters* **2010**, *10*, 5109–5115.

(22) Haro-González, P.; Martínez-Maestro, L.; Martín, I.; García-Solé, J.; Jaque, D. High-Sensitivity Fluorescence Lifetime Thermal Sensing Based on CdTe Quantum Dots. *Small* **2012**, *8*, 2652–2658.

(23) Zhao, H.; Vomiero, A.; Rosei, F. Ultrasensitive, Biocompatible, Self-Calibrating, Multiparametric Temperature Sensors. *Small* **2015**, *11*, 5741–5746.

(24) Liu, J.; Zhang, H.; Selopal, G. S.; Sun, S.; Zhao, H.; Rosei, F. Visible and Near-Infrared, Multiparametric, Ultrasensitive Nanothermometer Based on Dual-Emission Colloidal Quantum Dots. *ACS Photonics* **2019**, *6*, 2479–2486.

(25) Hsia, C.-H.; Wuttig, A.; Yang, H. An Accessible Approach to Preparing Water-Soluble $Mn^{2+}$-Doped (CdSSe)ZnS (Core)Shell Nanocrystals for Ratiometric Temperature Sensing. *ACS nano* **2011**, *5*, 9511–9522.

(26) McLaurin, E. J.; Vlaskin, V. A.; Gamelin, D. R. Water-Soluble Dual-Emitting Nanocrystals for Ratiometric Optical Thermometry. *Journal of the American Chemical Society* **2011**, *133*, 14978–14980.

(27) Park, Y.; Koo, C.; Chen, H.-Y.; Han, A.; Son, D. H. Ratiometric Temperature Imaging Using Environment-Insensitive Luminescence of Mn-Doped Core–Shell Nanocrystals. *Nanoscale* **2013**, *5*, 4944–4950.
